# Healthcare delivery modality and mental health–related intention to leave their job among nurses in Canada

**DOI:** 10.1371/journal.pmen.0000692

**Published:** 2026-09-15

**Authors:** Shauna Clayton, Safoura Zangiabadi, Hossam Ali-Hassan

**Affiliations:** 1 School of Kinesiology and Health Sciences, York University, Toronto, Ontario, Canada; 2 Glendon campus, York University, Toronto, Ontario, Canada; National University of Singapore, SINGAPORE

## Abstract

This study examines the association between healthcare delivery modality and Canadian nurses’ intentions to leave their job within two years due to stress, burnout, or mental health concerns during the COVID-19 pandemic. A secondary analysis was conducted on data from the cross-sectional Survey on Health Care Workers' Experiences During the Pandemic (SHCWEP), conducted by Statistics Canada between September and November 2021. The sample consisted of 3107 nurses, weighted to represent 353,980 Canadian nurses aged 18 and above. A multivariable logistic regression analysis was conducted to investigate the association between the mode of healthcare delivery (online, in-person, or blended) and the intention to exit the workforce due to mental health concerns, after adjusting for job, health-related, and sociodemographic factors. A desire to quit was reported by 33.5% of nurses, with 20.5% reporting mental health concerns as their reasoning. Online or blended modalities of care delivery yielded a decreased likelihood of reporting an intention to resign in comparison to in-person care delivery (OR =0.49, 95% CI: 0.45- 0.52; OR =0.81, 95% CI: 0.78- 0.84, respectively). Nurses are a critical asset to the medical field, and their retention impacts patient care directly and public health eventually. This study provides insight into nurses’ needs and can be informative for future implementation strategies that enhance existing training and education protocols and support nurses’ mental well-being.

## Introduction

After the World Health Organization’s (WHO) announcement of the Coronavirus 2019 (COVID-19) pandemic, the Canadian healthcare system underwent quick and drastic transformations [1]. Compared to previous outbreaks, such as the 2003 Severe Acute Respiratory Syndrome (SARS) and 2009 H1N1(Swine Flu) viruses, the impact of COVID-19 was unparalleled. High infection rates were associated with disproportionate staff-to-patient ratios, subsequently causing an unprecedented strain on medical resources. Efforts to optimize patient care and protect healthcare workers included adapting existing standards of care to incorporate physical distancing protocols. This included adopting telemedical procedures, including telephone, video conferencing, and text messaging, in place of in-person visits for most non-urgent matters [2]. Data collected in the Fall of 2021 showed that 24% of healthcare workers incorporated telemedical protocols into their practice during the pandemic, including telephone (87%), video (47%), and email and/or instant messaging (26%) [3]. These adaptations allowed medical staff to triage, assess, consult, diagnose, and remotely monitor patients with medical concerns [4]. Decision-making around such policies aimed to implement urgent public health recommendations while ensuring continued access to patient healthcare. Although such changes were deemed beneficial in the broader sense of mitigating infections, the impromptu changes posed challenges to the healthcare infrastructure. Lack of preparedness and ethical considerations, including the inadequate assessment and treatment of patients virtually, were among the concerns expressed by medical professionals during the pandemic [5]. Moreover, complications with virtual care, including usability issues and workflow disruptions, also presented challenges [6]. Some medical professionals reported difficulty incorporating their clinical background into a telemedical framework due to a lack of training and the sudden and urgent implementation of these protocols [5]. A 2024 systematic review comparing the effectiveness of telehealth with in-person care during the COVID-19 pandemic found that telehealth achieved outcomes comparable to those of in-person care across a wide range of clinical settings and health outcomes. As healthcare systems continue to evolve, ongoing evaluation of telehealth will remain essential [7].

Many organizations, such as the WHO, acknowledged the implications of COVID-19 on mental health, highlighting frontline workers as some of the most at-risk populations [8]. These findings were observed throughout the initial stages of the pandemic and were further substantiated by the increasing amount of data as it progressed over several years. A prospective cohort study conducted in March-May 2020 found that nurses who reported a high fear of infection towards themselves or others had increased mental health symptoms associated with depression, anxiety, and stress [9]. In another example, a systematic review and meta-analysis of data collected on nurses’ mental health during the pandemic found that these workers reported higher occurrences of anxiety, stress, depression, and insomnia compared to reports collected from the general population [10]. Historically, nurses have been at the forefront of direct client care, including the clinical assessment and treatment of medical issues. Data collected during the pandemic aligns with pre-pandemic reports on nurses’ mental health, indicating risks of burnout and psychological distress, including stress, depression, and anxiety [11,12]. Recent meta-analyses indicate that burnout across multiple dimensions remains higher among nurses than in the pre-pandemic period and is higher than among other healthcare workers [12]. Nurse burnout is prevalent worldwide and is often characterized by a diminished sense of personal accomplishment. It has been linked to numerous workplace and personal factors, including unmanageable workloads, poor work–life balance, inadequate workplace support during difficult situations such as severe patient illness and death, role conflict, negative emotions, moral distress, stress, family problems, long commuting distances, unpredictable work tasks, and limited opportunities for career advancement [13,14].

The global nursing shortage was a well-recognized challenge before the COVID-19 pandemic. Evidence indicates that the pandemic further exacerbated workforce shortages and negatively affected nurse retention [15]. Findings from the pandemic period suggest that increased pressures on healthcare systems amplified pre-existing workplace stressors among nurses. Compared with other healthcare professionals, nurses reported particularly high levels of psychological distress [16].

Staffing shortages, increased workloads, lack of personal protective equipment (PPE), social isolation, and fear of viral exposure exacerbated an already stressful work environment for nurses [17]. Nurses also reported concerns regarding workplace safety, inadequate training, burnout, and working within healthcare systems that were heavily strained and underprepared [18]. A 2025 review of pre- and post-pandemic evidence found increased nurse turnover intentions, particularly among those caring for patients with COVID-19. Contributing factors included fear of infection, concerns about future pandemics, inadequate preparation, shortages of resources and personal protective equipment (PPE), poor workplace safety climate, COVID-19-related stigma or discrimination, and insufficient organizational support, with nurses feeling their concerns were not adequately acknowledged or addressed. [19]. Although much of the literature has focused on pandemic-related stressors and mental health outcomes among frontline healthcare workers, workforce retention and healthcare system sustainability remain ongoing concerns. Understanding organizational and healthcare delivery factors associated with nurses’ mental health, burnout, and intentions to leave their job may help inform workforce retention and health system resilience strategies. To date, little is known about how different modes of healthcare delivery may influence nurses’ mental health and intentions to leave the workforce.

The purpose of this study was to investigate the relationship between the mode of healthcare delivery and nurses’ intention to exit the workforce within two years due to stress, burnout, or mental health during the COVID-19 pandemic.

## Methods

The study was a secondary analysis of data collected by Statistics Canada using the Health Care Workers’ Experiences During the Pandemic Survey (SHCWEP) between September and November 2021 [16]. The goal of SHCWEP was to gain insight into how COVID-19 may have impacted healthcare workers in Canada. A total of 12,246 healthcare workers aged 18 years or older residing in Canada completed the study.

The dependent variable for this study was nurses’ intention to leave their jobs due to stress, burnout, or mental health challenges. The survey first asked each participant to report an approximation of the duration of their current employment. Following the initial question about employment, those who reported an intention to leave the workforce within two years were asked to respond to questions about factors that impacted their decision-making. Participants who reported reasoning to quit being stress, burnout, or mental health, were classified as “yes,” and all other participants were classified as “no.” The main independent variable was the mode of service provision, which was categorized into three options: in-person, online through video, email, text, or instant messaging, and blended, which was an integration of both modalities.

The study adjusted for several covariates, which included job-related, sociodemographic, and health-related factors. Job-related factors included years worked in the job (<10 years, 10–19 years, > 20 years), number of locations worked at (one location, multiple locations), type of healthcare job location (acute care, outpatient and ambulatory care, other) and whether they received formal training on IPC (yes, no). Sociodemographic and health-related factors included gender (male, female), age (18–34, 35–44, 45–54, > 55years), province of residence (Atlantic provinces, Quebec, Ontario, Manitoba, Saskatchewan, Alberta, British Columbia), household income (≤$149,999, ≥ 150,000, not stated), immigration status (non-immigrant, immigrant or non-permanent resident) and self-perceived overall health (Poor/fair, good, very good/excellent).

Relationships between service modality, the covariates and the main dependent variable were assessed using chi-square and a bivariate logistic regression model. Following these initial analyses, a multivariable logistic regression analysis was utilized to examine the relationship between service modality and intention to quit while controlling for all covariates. Population weights and bootstrapping techniques were employed to adjust for the complex survey sampling methodology. The 95% confidence intervals were based on the resulting design-based standard errors and for several parameter estimates, these standard errors were relatively small, resulting in correspondingly narrow confidence intervals. Missing data were minimal (<5% for all variables). Therefore, complete-case (case deletion) analysis was used. Multicollinearity was assessed using variance inflation factors (VIFs). Model fit was evaluated using the Omnibus, Cox & Snell, and Nagelkerke goodness-of-fit tests. Interaction terms were not examined, as the primary objective was to estimate the overall association between mode of healthcare delivery and nurses’ intentions to leave their job. All statistical analyses were performed using the Statistical Package for the Social Sciences (SPSS, version 28.0). Significance was set at a p < 0.05.

Ethical review by the University Ethics Board was not necessary for this study, as the SHCWEP public use microdata files generated by Statistics Canada are openly available and are suitably safeguarded by legal measures, such as those outlined in the Data Liberation Initiative [4].

## Results

This study included 3107 nurses, weighted to represent 353,980 nurses in Canada. Table 1 presents descriptive statistics regarding the intention to exit the workforce within two years and associated variables. Analyses showed that 33.5% of nurses reported an intention to leave their job, and 20.5% reported stress, burnout, or mental health concerns as factors influencing their decision-making. A descriptive summary of characteristics and the bivariate and multivariable relationships between service modality and job-, sociodemographic-, and health-related variables with intention to quit due to stress, burnout, or mental health are reported in Table 2. Most participants reported living in Ontario at the time of survey completion (36.6%), and most were female (89.8%). Additionally, 32% of nurses were in the 18–34 age category, 61.8% reported working in an acute care setting, and 56.2% reported an income of ≤$149,999. Regarding service modality, 85.7% reported working in person, 3.6% worked online, and 10.6% provided blended healthcare services.

**Table 1:** Intention and reasons for quitting current job within two years among nurses in Canada - weighted estimates based on 3,107 respondents, representing approximately 353,980 nurses.

Factor	Number	(%)
Intention to quit current job within 2 years		
Yes	118,627	33.5
No	235,352	66.5
Reasons for quitting job*		
Mental health concerns	72,396	20.5
Retiring	43,255	12.2
Lack of job satisfaction	47,611	13.5
Concerns physical health	35,957	10.2
Concerns about household members	21,321	6.0
Financial impacts	9,425	2.7
Health care system	35,908	10.1
Other career opportunity	32,943	9.3
Other	13,607	3.8

*Percentages may not add up to 100 because of missing data.

**Table 2:** Characteristics of participants and associations with quitting job due to mental health reasons among nurses in Canada.

Factor	%^*^	Crude OR	95% CI	p-value	Adjusted OR	95% CI	p-value
Job-related factors							
Mode of healthcare delivery							
In-person	85.7	1			1		
Online	3.6	0.53	0.50, 0.55	<0.001	0.49	0.45, 0.52	<0.001
Blended	10.6	0.57	0.55, 0.59	<0.001	0.81	0.78, 0.84	<0.001
Years worked in the job							
Less than 10 years	42.5	2.79	2.73, 2.86	<0.001	2.30	2.22, 2.39	<0.001
10 to 19 years	25.6	1.35	1.32, 1.39	<0.001	1.77	1.71, 1.84	<0.001
20 years or more	25.6	1			1		
Not stated	6.4	1.54	1.48, 1.60	<0.001	1.70	1.61, 1.79	<0.001
Number of locations worked at							
One location	72.0	1			1		
Multiple locations	25.1	0.85	0.84, 0.87	<0.001	0.94	0.92, 0.96	<0.001
Type of healthcare job location							
Acute care	61.8	1			1		
Outpatient and ambulatory care	11.7	0.34	0.33, 0.35	<0.001	0.37	0.35, 0.38	<0.001
Other^#^	24.3	0.85	0.83, 0.86	<0.001	0.99	0.97, 1.01	0.414
Received formal training on IPC							
Yes	90.7	1			1		
No/ Workplace do not have an IPC policy/valid skip	8.7	1.74	1.69, 1.78	<0.001	2.66	2.57, 2.74	<0.001
Socio-demographic and health related factors							
Gender							
Male	10.1	1			1		
Female	89.8	0.92	0.89, 0.94	<0.001	1.04	1.00, 1.08	0.065
Age							
18 to 34 years	32.0	1			1		
35 to 44 years	24.8	0.31	0.31, 0.32	<0.001	0.36	0.35, 0.37	<0.001
45 to 54 years	21.5	0.23	0.22, 0.24	<0.001	0.39	0.37, 0.40	<0.001
55 years and older	21.5	0.41	0.40, 0.42	<0.001	0.85	0.82, 0.88	<0.001
Province of residence							
Atlantic provinces	8.5	2.36	2.27, 2.44	<0.001	1.90	1.82, 1.97	<0.001
Quebec	22.9	1			1		
Ontario	36.6	3.31	3.22, 3.40	<0.001	2.75	2.68, 2.84	<0.001
Manitoba	3.9	2.70	2.58, 2.83	<0.001	2.73	2.58, 2.88	<0.001
Saskatchewan	3.2	2.11	2.00, 2.23	<0.001	1.82	1.72, 1.94	<0.001
Alberta	12.4	2.53	2.45, 2.61	<0.001	2.52	2.43, 2.62	<0.001
British Columbia	12.4	3.48	3.37, 3.59	<0.001	3.37	3.26, 3.49	<0.001
Household income							
≤$149,999	56.2	1.27	1.25, 1.30	<0.001	0.99	0.97, 1.01	0.364
≥ 150,000	28.3	1			1		
Not stated	15.5	1.19	1.16, 1.22	<0.001	1.14	1.10, 1.18	<0.001
Immigration status							
Non-immigrant	74.8	1			1		
Immigrant or non-permanent resident	22.5	0.51	0.50, 0.52	<0.001	0.39	0.38, 0.40	<0.001
Perceived general health							
Poor/Fair	12.5	1			1		
Good	36.7	0.46	0.45, 0.48	<0.001	0.46	0.45, 0.47	<0.001
Very good/Excellent	50.7	0.33	0.32, 0.33	<0.001	0.30	0.30, 0.31	<0.001

* Percentages may not add up to 100 because of missing data.

# Long-term care and Community/home care.

For the multivariable logistic regression, no evidence of problematic multicollinearity was observed. The overall model was statistically significant (Omnibus χ²(24) = 48,583.357, p < 0.001) and the pseudo-R^2^ values were Cox & Snell R^2^ = 0.138 and Nagelkerke R^2^ = 0.216. Results of the model yielded a significant relationship between the mode of healthcare service delivery and the intention to exit the workforce due to mental health concerns. Participants indicating an online or blended service modality had a decreased likelihood of reporting an intention to quit due to stress, burnout, or mental health compared to nurses working in-person (OR =0.49, 95% CI: 0.45- 0.52; OR =0.81, 95% CI: 0.78- 0.84, respectively). Among job-related factors, nurses working in the field for less than ten years appeared 2.3 times more likely to report an intention to quit than those with twenty or more years of experience. Working at multiple locations yielded a decreased likelihood of reporting an intention to exit the field compared to nurses working at one location. In addition, individuals who indicated that they did not receive formal Infection Prevention and Control (IPC) training were also more likely to report an intention to resign than those who reported receiving the training (OR= 2.66, 95% CI: 2.57- 2.74).

Nurses reporting excellent or very good general health had lower odds of reporting an intention to quit than nurses with fair or poor self-perceived general health (OR= 0.30, 95% CI: 0.30- 0.31).

## Discussion

Periods of healthcare system strain were associated with substantial changes in healthcare delivery models across Canada, including the increased use of virtual care approaches intended to optimize patient care and reduce pressure on healthcare resources. The present study examined the relationship between the mode of healthcare delivery and nurses’ intention to leave the workforce within the next two years due to stress, burnout, or mental health issues. Intention to quit was reported by 33.5% of nurses, with 20.5% citing stress, burnout, or mental health issues as their reason. Nurses who engaged in virtual or blended healthcare delivery modalities had decreased odds of reporting an intention to quit compared to those who solely worked in person (OR =0.49, 95% CI: 0.45- 0.52; OR =0.81, 95% CI: 0.78- 0.84, respectively). The results of this study highlight the importance of strategizing ways to optimize mental well-being among frontline medical workers, such as nurses, within a healthcare framework. Telemedicine, or incorporating a blended modality, may be an approach that could be positively associated with nurses’ experiences in the workforce.

The present study extends previous work by addressing a distinct research question regarding the role of mental health in workforce retention. Although prior analyses using the same national dataset showed that physicians and nurses providing virtual or blended care were less likely to report intentions to quit, those studies focused primarily on job dissatisfaction or examined physicians as a separate professional group [20,21]. In contrast, the present study specifically examines intention to quit driven by stress, burnout, and mental health, allowing us to isolate the psychological pathway underlying workforce attrition. This distinction is important because interventions aimed at improving job satisfaction (e.g., workplace organization or compensation) differ from those needed to address burnout and psychological distress (e.g., mental health supports, workload management, and resilience-promoting organizational strategies). Furthermore, our findings demonstrate that 20.5% of nurses reported mental health-related intentions to quit—nearly three times the proportion previously reported among physicians (7.5%)—highlighting the disproportionate mental health burden experienced by nurses during the COVID-19 pandemic and underscoring the need for profession-specific retention strategies. Together, these findings identify flexible care delivery modalities as potentially relevant workplace characteristics associated with nurses’ psychological and professional experiences under stress, while also highlighting that nurses face a substantially more acute mental health crisis than their physician counterparts. However, given the cross-sectional design, the observed association should not be interpreted as evidence that telemedical flexibility buffers negative impacts on mental well-being or contributes to workforce retention. Rather, these findings generate hypotheses about potentially beneficial workplace characteristics associated with virtual or blended care that can be examined in longitudinal or intervention-based research.

Previous research has shown that healthcare professionals experienced substantial physical and emotional exhaustion, which are core symptoms of burnout, as well as other mental health challenges including depression and anxiety [22]. In addition, the COVID-19 pandemic introduced added responsibilities related to preventive and treatment protocols aimed at optimizing patient care. Nurses were among the healthcare professionals most exposed to the virus, as well as to quarantine mandates and social stigma [23]. Research conducted during the pandemic suggests that burnout among nurses was associated not only with workload, but also with fear of viral exposure [24]. However, some studies reported lower burnout among nurses who incorporated virtual protocols into their practice, particularly among female nurses [25]. Although healthcare professionals initially expressed concerns regarding telemedicine and clinical practice, studies also reported perceived benefits including improved efficiency, productivity, and job satisfaction [26], and positive impact on work–life balance and burnout [27].

Job-related factors, including the number of healthcare settings worked at, the number of years on the job, and Infection Prevention and Control (IPC) training, were also associated with the intention to quit due to stress, burnout, or mental health issues. Individuals with ten years of experience or less appeared 2.3 times more likely to report this outcome than those with more experience. Thus, newer nurses who lack training compared to their more experienced colleagues may still need to develop strategies for coping with high-stress situations. Moreover, the pandemic prompted the deployment of impromptu protocols and adaptations to standard clinical practice, which may be associated with high amounts of stress and uncertainty. During this time, nurses were tasked with being at the forefront of clinical care and companionship for many severely ill and dying patients who were not allowed to be among family due to an expansion of physical distancing protocols that implemented strict no-visitation policies. This may have been particularly challenging for nurses who lacked in experience and exposure to these circumstances in general.

Nurses who worked in multiple settings reported a lower likelihood of wanting to resign due to stress, burnout, or mental health issues compared to those who worked in just one setting. Working in multiple settings may have contributed to this outcome by providing variations in day-to-day experiences. One study examining shift characteristics associated with burnout among nurses in the United Kingdom and Ireland found that nurses with less choice over the duration of their shifts, as well as those experiencing staffing shortages, were more likely to experience burnout [28]. Another study examining healthcare workers’ perceptions and satisfaction with telemedicine found that participants did not find telemedicine monotonous and reported better job satisfaction [29]. Multiple settings may also increase opportunities for network expansion and team building, which has been associated with lower burnout and work-related stress [30].

Lack of IPC training was another factor significantly associated with nurses’ intention to exit the workforce due to stress, burnout, or mental health. IPC training educates medical professionals on safe workplace practices that can help reduce the spread of disease and illness. The training provides a framework for emergency preparedness, triage strategies, risk screening, and quarantine protocols [17]. Periods of healthcare system strain created substantial pressure on healthcare resources and highlighted the need for effective infection prevention and control (IPC) practices. During this period, IPC training may have provided healthcare workers with a structured framework for day-to-day clinical practice and may have helped reduce fears associated with viral exposure, which have been linked to burnout and stress [31].

Sociodemographic results indicated that gender was not significantly associated with intention to quit in the adjusted analysis. Although the present sample had more females than males, which is a good representation of the nursing field in general [32], this finding contrasts with previous research showing females working in the medical field may be more susceptible to burnout and mental health challenges compared to males [33]. Literature on these topics has attributed this susceptibility to emotional exhaustion and challenges faced with work-life balance [34]. In addition, nurses older than 34 years old appeared to have been at a decreased risk of wanting to quit their jobs compared to nurses aged 18–34. These findings could be explained by other data collected in this study, which showed an association between years spent on the job and intention to exit the workforce, with fewer years yielding an increased likelihood of this outcome. Younger nurses, in general, may have fewer coping strategies and less resilience for high-stress situations that might buffer against adverse mental health effects that could impact one’s desire to remain working. In addition, immigrants, compared to non-immigrants, had a decreased likelihood of reporting an intention to quit due to stress, burnout, and mental health. Immigration is a complex and competitive process that might be associated with resilience and adaptive coping strategies [35] and could potentially explain why these individuals were at a decreased risk.

Finally, those who reported their general health as either good or excellent had decreased odds of reporting an intention to quit their job compared to those who reported poor general health. Poor general health has been shown to increase susceptibility to burnout and mental health symptoms such as stress and depression. Poor health may impact general functioning and result in an impaired ability to perform work-related tasks. Furthermore, public health organizations, including the WHO, declared those with health issues to be among the most at-risk for severe COVID-19 symptoms and potential death [8]. Constant exposure to COVID-19 and general fear of the virus may have, therefore, contributed to nurses with poor general health and increased mental and physical exhaustion, perpetuating feelings of stress and burnout and other mental health symptoms, leading to a desire to exit the workforce.

### Limitations

This study was the first to examine the relationship between the mode of healthcare delivery and nurses’ intention to quit their jobs within two years due to stress, burnout, and mental health. However, the cross-sectional design does not allow for establishing temporal relationships or causal inferences. Consequently, the possibility of reverse causation cannot be excluded, as nurses’ existing levels of stress, burnout, or mental health concerns may have influenced the type of healthcare delivery in which they were working. In addition, this study has the potential for residual confounding. Although the analyses adjusted for several occupational, sociodemographic, and health-related characteristics, the survey did not capture some potentially important factors, including pre-existing mental health conditions or psychological vulnerability, stressors within the home environment, and workplace characteristics such as patient acuity, job responsibilities, schedule flexibility, and other aspects of the work environment. These unmeasured factors may have influenced both the mode of healthcare delivery and nurses’ intentions to leave the workforce. Therefore, the observed associations should be interpreted with caution. Another limitation is that healthcare delivery mode was measured using broad categories (in-person, online, and blended), which likely encompassed heterogeneous models of care (e.g., telephone, video, and asynchronous communication). The available data did not allow for more detailed classification, and this may have resulted in some exposure misclassification.

## Conclusion

This study examined the association between healthcare delivery modality and nurses’ intentions to leave their job due to stress, burnout, and mental health concerns. Findings suggest that blended or virtual models of healthcare delivery were associated with a lower likelihood of quitting intentions. These results support existing literature that telemedicine is associated with more positive workplace experiences among healthcare providers. Because this study used a cross-sectional design, these findings do not demonstrate that changing delivery modality would improve retention. Rather, the findings generate hypotheses about potentially beneficial workplace characteristics associated with virtual or blended care that warrant further investigation in longitudinal or intervention-based research. Understanding organizational and healthcare delivery factors associated with nurse wellbeing and retention remains important for supporting workforce sustainability, a resilient healthcare system, and an improved public health.
