# Supplementary material for: Healthcare delivery modality and mental health–related intention to leave their job among nurses in Canada
Source: PLOS Ment Health. 2026 Sep 15;3(9):e0000692. doi: 10.1371/journal.pmen.0000692 (PMC13577573; doi:10.1371/journal.pmen.0000692)
Supplement: Supplementary file 1 [file pmen.0000692.s002.docx]

**Response to Reviewers**

We thank the three reviewers for their thoughtful and extensive comments. We have revised the manuscript to address all of their feedback, which has significantly strengthened the paper. Please find below our response to each comment.

**Reviewer #1 Comments:**

I recommend this manuscript to be Major Revision, some comments need the author below address:

**1.** The authors should consistently use causal-neutral language such as "was associated with" rather than "led to," "reduced" or "contributed to".

**Response:** Thank, you for your suggestion. Throughout the manuscript we replaced terms and expressions that could indicate a causality or positive impact with more causal-neutral language.

**2**. The limitations section should discuss the potential impact of residual confounding more explicitly

**Response:** We have expanded the Limitations section to acknowledge the possibility of residual confounding.

**3**. Please double check, how the complex survey design was incorporated into the regression analyses

**Response:** In the regression analysis, population weights and bootstrapping techniques were employed to adjust for the complex survey sampling methodology.

**4**. The lower odds of intending to leave among nurses providing online or blended care may reflect differences in workplace characteristics rather than the delivery modality itself. For example, nurses providing virtual care may have lower patient acuity, different job responsibilities, greater schedule flexibility or better organizational support. These alternative explanations should be acknowledged in the Discussion as well.

**Response:** Thank you for the comment. We have revised the Limitations section to acknowledge that unmeasured workplace characteristics (e.g., patient acuity, job responsibilities, and schedule flexibility) may have influenced the observed associations, and therefore residual confounding cannot be ruled out.

**5**. Please consider including more recent literature (2023-2025) on nurse burnout, workforce retention and post-pandemic implementation of virtual care to better contextualize the findings.

**Response:** Thank you for your suggestion. We added recent references (2024 and 2025) to the paper, namely [1], [2], [3], [4], [5], and [6].

**Reviewer #2 Comments:**

Strengths:

1. Large nationally representative dataset.

2. Timely and policy-relevant research question.

3. Appropriate use of multivariable regression.

4. Important workforce mental health outcome.

5. Findings have practical relevance for health workforce planning.

**Response:** Thank you for pointing out the strengths of the study.

**Areas for improvement:**

**1**. The Introduction section is generally well organized. However, more literature on pre-pandemic nurse retention would improve context, and recent post-pandemic workforce evidence could also be incorporated.

**Response:** Thank you for your suggestion. We added recent references to the paper that would improve context, namely [1], [2], [3], [4], [5], and [6].

**2**. The authors should better justify the novelty of the study, given their previous publications using the same dataset on physicians and nurses (reference #15 and #16). The manuscript should clearly articulate the additional contribution of focusing specifically on mental health-driven intention to quit.

**Response:** Thank you for your suggestion. The distinction between this study and the two previous ones is now clearer and the contribution of the current paper, by focusing on mental health-driven intention to quit, is more explicit.

**3**. The Methods section:

**a.** Would benefit from additional detail on the handling of missing data, proportion of missing values, complete-case versus imputation, model diagnostics, assessment of multicollinearity, goodness-of-fit, and whether interaction terms were examined. These are standard expectations for logistic regression reporting.

**Response:** Thank you for this suggestion. We have revised the Statistical Analysis section to clarify the handling of missing data, assessment of multicollinearity and model fit, and to indicate that interaction terms were not examined because the study aimed to estimate the overall association between mode of healthcare delivery and nurses' intentions to leave the workforce.

**b.** Should explain the very narrow confidence intervals reported for several adjusted odds ratios e.g., Online: OR = 0.49, 95% CI 0.45–0.52; IPC: OR = 2.66, 95% CI 2.57–2.74; and Ontario: OR = 2.75, 95% CI 2.68–2.84. Such precision is unusual given an effective sample of approx. 3,100 nurses.

**Response:** Thank you for this observation. The reported confidence intervals are those produced by the survey-weighted logistic regression analyses using the Statistics Canada sampling weights together with the bootstrap weights to account for the complex survey design. We have verified that the reported confidence intervals are correct and reflect the design-based variance estimation implemented in SPSS.

**4**. Results are clearly presented.

**a.** However, the narrative largely repeats Table 2. Consider focusing on strongest predictors, unexpected findings and clinically important associations.

**Response:** Thank you for this suggestion. We have revised the Results section to reduce repetition of the findings presented in Table 2 and to improve the overall flow.

**b.** Healthcare delivery modality is categorized simply as in-person, online and blended. However, these categories likely encompass tremendous heterogeneity e.g., telephone only, video consultation, asynchronous messaging, occasional virtual clinics and predominantly virtual practice. The manuscript should acknowledge that the exposure is relatively crude.

**Response:** Thank you. We have acknowledged this limitation in the Discussion section.

**5.** The interpretation of the findings should be more cautious.

**a**. The study is cross-sectional, causal language should be avoided (e.g., “telemedicine may contribute positively to nurses' experiences” and “blended care contributes to workforce retention”, etc).

**Response:** Thank, you for your suggestion. Throughout the manuscript we replaced terms and expressions that could indicate a causality or positive impact with more causal-neutral language.

**b**. The possibility of reverse causation (e.g., nurses already experiencing burnout may avoid virtual work) and residual confounding should be discussed more explicitly.

**Response:** Thank you for this important comment. We have revised the Limitations section to acknowledge that.

**c.** Policy recommendations regarding telemedicine and workforce retention should be framed as hypotheses supported by associations rather than causal conclusions.

**Response**: We replaced terms and expressions that could indicate causality with more causal-neutral terms like “association”.

**6**. The limitations section should be expanded to acknowledge important unmeasured confounders (e.g., nursing specialty, workload, staffing levels, resilience, and burnout severity, etc) and the inability to establish temporal relationships.

**Response:** Thank you for this comment. This has been addressed in response to a previous comment.

**Reviewer #3 Comments:**

The study talks about associations between the mode of employment (remote, hybrid, and presential) and other characteristics, and how nurses wanted to leave the job due to mental health problems. It is an important topic, and the paper was well written. However, I still have some questions to better understand and improve the work.

The title is confusing; I could not understand the paper's message from it. A simpler and more direct title would be better.

**Response:** Thank you for pointing that out. The title of the paper is now shorter and simpler.

There are some acronyms not spelled out in the first appearance.

**Response:** Acronyms in the paper, like “SARS” are now spelled out at their first appearance.

"A total of 12,246 healthcare workers aged 18 years or older residing in Canada completed the study." So why "This study included 3107 nurses, weighted to represent 353,980 nurses in Canada."

Does Table 1 represent the 353,980 nurses?

**Response**: The present study was restricted to the subset of 3,107 respondents who identified as nurses. Survey weights were applied so that these respondents represented an estimated 353,980 nurses in Canada. This has been added to the title of Table 1.

**REFERENCES**

1.	Hatef E, Wilson RF, Zhang A, Hannum SM, Kharrazi H, Davis SA, et al. Effectiveness of telehealth versus in-person care during the COVID-19 pandemic: a systematic review. npj Digital Medicine. 2024;7(1):157.

2.	Zheng J, Feng S, Feng Y, Wang L, Gao R, Xue B. Relationship between burnout and turnover intention among nurses: a network analysis. BMC nursing. 2024;23(1):921.

3.	Getie A, Ayenew T, Amlak BT, Gedfew M, Edmealem A, Kebede WM. Global prevalence and contributing factors of nurse burnout: an umbrella review of systematic review and meta-analysis. BMC nursing. 2025;24(1):596.

4.	Farahani MA, Nargesi S, Saniee N, Dolatshahi Z, Heidari Beni F, Shariatpanahi S. Factors affecting nurses retention during the COVID‐19 pandemic: a systematic review. Human Resources for Health. 2024;22(1):78.

5.	Buckley L, McGillis Hall L, Price S, Visekruna S, McTavish C. Nurse retention in peri-and post-COVID-19 work environments: a scoping review of factors, strategies and interventions. BMJ open. 2025;15(3):e096333.

6.	Sicorschi Gutu C, Schmitz-Grosz K, Sommer-Meyer C, Niklewski G. Physicians’ Perspectives, Well-being, and Fulfilment in Telemedicine: A Cross-Sectional Study. Telemedicine Reports. 2024;5(1):tmr. 2024.0005.
