# Supplementary material for: Healthcare delivery modality and mental health–related intention to leave their job among nurses in Canada
Source: PLOS Ment Health. 2026 Sep 15;3(9):e0000692. doi: 10.1371/journal.pmen.0000692 (PMC13577573; doi:10.1371/journal.pmen.0000692)
Supplement: Supplementary file 2 [file pmen.0000692.s003.docx]

# Response to Reviewers

We thank all reviewers for their valuable feedback. We have addressed all your comments and modified the manuscript accordingly. Our response to each comment can be found below.

**Journal Requirements:**

If the reviewer comments include a recommendation to cite specific previously published works, please review and evaluate these publications to determine whether they are relevant and should be cited. There is no requirement to cite these works unless the editor has indicated otherwise.

Please review your reference list to ensure that it is complete and correct. If you have cited papers that have been retracted, please include the rationale for doing so in the manuscript text, or remove these references and replace them with relevant current references. Any changes to the reference list should be mentioned in the rebuttal letter that accompanies your revised manuscript. If you need to cite a retracted article, indicate the article’s retracted status in the References list and also include a citation and full reference for the retraction notice.

**Response: The reviewers did not recommend to cite a specific published work. There are no changes to the reference list.**

**Reviewer #1:** So great that you have improved your manuscript so far, I recommend this manuscript subject to these relatetively minor correction, I believe the manuscript would be suitable for publication. From the minor revision, there a few issues remain before it suitable for accepatance.

**Response: Thank you for your assessment.**

1. First, please recheck Table 2, the adjusted estimate for outpatient/ambulatory care (OR = 0.37, 95% CI 0.35–0.38) is reported with p = 0.366; the confidence interval and p-value appear statistically inconsistent.

**Response:** **Thank you for carefully identifying this inconsistency. We rechecked Table 2 against the original SPSS output and confirmed that a few values were inadvertently placed in the incorrect columns during transcription. We apologize for this mistake. The corrected estimates are outpatient/ambulatory care: OR = 0.37 (95% CI: 0.35–0.38), p < 0.001; Other care: OR = 0.99 (95% CI: 0.97–1.01), p = 0.414; and female: OR = 1.04 (95% CI: 1.00–1.08), p = 0.065. These values have been corrected in Table 2 and throughout the manuscript [Page 16]. We also rechecked all remaining values in Table 2 against the original SPSS output and confirmed their accuracy. Thank you again for pointing this error out.**

2. Second, the Discussion states that nurses with “two years of experience or less” had 2.3-fold higher odds of the outcome, whereas the analyzed category is “less than 10 years.” Please correct this discrepancy.

**Response: We apologize for this mistake. The correct number of years is ten as you indicated. Thank you for pointing this out.**

3. Should you add the statements suggesting that virtual care may confer psychological benefits should be further tempered given the cross-sectional design.

**Response: Thank you for pointing that out. We have updated the Discussion and Conclusion sections as suggested by Reviewer 2 to remove any potential reference to a causal relation.**

**Reviewer #2:** The authors have responded constructively to the comments and have made several substantive improvements to the manuscript.

**Response: Thank you for your assessment.**

However, a few issues remain:
1. The explanation for the unusually narrow confidence intervals is not fully convincing and should be further clarified, particularly regarding the complex survey variance estimation i.e., authors have explained how the CIs were generated but not convincingly why they are so narrow.
**Response: Thank you for this important comment and for the opportunity to further clarify this point. SHCWEP included four healthcare-worker groups, and the present analysis was restricted to nurses resulting in an unweighted sample of 3,107 nurses, representing 353,980 Canadian nurses after application of the Statistics Canada sampling weights. Sampling weights were used to obtain population-representative estimates, while bootstrap weights were used for design-based variance estimation to account for the complex survey design. Accordingly, the width of the 95% confidence intervals was determined by the resulting design-based standard errors rather than by the unweighted sample size alone. For several of the associations highlighted, the bootstrap variance estimation yielded relatively small standard errors resulting in precise parameter estimates and correspondingly narrow 95% confidence intervals. We have further clarified this point in the Methods section of the revised manuscript [Page 7]. We appreciate the reviewer’s thoughtful comment and the opportunity to clarify this aspect of the analysis.**

2. Although the authors state that causal language has been removed, some causal or causal-suggestive wording remains in the Discussion (e.g., flexible modalities “may confer psychological as well as professional benefits” and that telemedical flexibility “may help buffer” negative impacts and “may potentially contribute” to retention) and Conclusion (telemedicine may “help optimize patient care while contributing to more positive workplace experiences”) and should be revised to reflect the cross-sectional design.

I suggest the authors to explicitly state that the findings generate hypotheses about potentially beneficial workplace characteristics associated with virtual/blended care, rather than demonstrating that changing delivery modality will improve retention.

**Response: thank you for your suggestion. We have updated the Discission and Conclusion sections as proposed.**

3. There appear to be serious inconsistencies in Table 2 p-values. For example, the table reports:
a. Outpatient/ambulatory care: adjusted OR 0.37, 95% CI 0.35–0.38, p = 0.366;
b. Other care: adjusted OR 0.99, 95% CI 0.97–1.01, p = 0.990; and
c. Female: adjusted OR 1.04, 95% CI 1.00–1.08, p < 0.001.

These combinations are questionable. In particular, an OR of 0.37 with a 95% CI of 0.35–0.38 cannot plausibly have p = 0.366, because the CI is far from 1. Likewise, OR 0.99 with CI 0.97–1.01 would not normally correspond to p = 0.990. The female estimate also warrants checking. The table should therefore be rechecked against the original statistical output, not merely copy-edited.

**Response:** **Thank you for carefully identifying these inconsistencies. We appreciate the opportunity to clarify and correct these values. We rechecked Table 2 directly against the original output and confirmed that a few values were inadvertently placed in the incorrect columns during transcription. We apologize for this mistake. The corrected estimates are outpatient/ambulatory care: adjusted OR = 0.37 (95% CI: 0.35–0.38), p < 0.001; Other care: adjusted OR = 0.99 (95% CI: 0.97–1.01), p = 0.414; and female: adjusted OR = 1.04 (95% CI: 1.00–1.08), p = 0.065. These corrections have been made in Table 2 and throughout the manuscript [Page 16]. We also carefully rechecked all remaining values in Table 2 against the original SPSS output and confirmed their accuracy. Thank you again for pointing this error out.**

**Reviewer #3:** I thank the authors for their response to the other reviewers and me.

**Response: Thank you for your assessment.**
