# Supplementary material for: Healthcare delivery modality and mental health–related intention to leave their job among nurses in Canada
Source: PLOS Ment Health. 2026 Sep 15;3(9):e0000692. doi: 10.1371/journal.pmen.0000692 (PMC13577573; doi:10.1371/journal.pmen.0000692)
Supplement: Peer review history

# Peer review: Decision Letter 0

**DOI:** 10.1371/journal.pmen.0000692.r001

**Authors:** Lambert Zixin Li

10 Jul 2026

PMEN-D-26-00345

Mental Health as a Driver of Nurses’ Intention to Leave: The Role of Healthcare Delivery Modality During System Disruption in Canada

PLOS Mental Health

Dear Dr. Ali-Hassan,

Thank you for submitting your manuscript to PLOS Mental Health. After careful consideration, we feel that it has merit but does not fully meet PLOS Mental Health’s publication criteria as it currently stands. Therefore, we invite you to submit a revised version of the manuscript that addresses the points raised during the review process.

Please submit your revised manuscript by Sep 08 2026 11:59PM. If you will need more time than this to complete your revisions, please reply to this message or contact the journal office at mentalhealth@plos.org. When you're ready to submit your revision, log on to https://www.editorialmanager.com/pmen/ and select the 'Submissions Needing Revision' folder to locate your manuscript file.

Please include the following items when submitting your revised manuscript:

- A letter that responds to each point raised by the editor and reviewer(s). You should upload this letter as a separate file labeled 'Response to Reviewers'.
- A marked-up copy of your manuscript that highlights changes made to the original version. You should upload this as a separate file labeled 'Revised Manuscript with Track Changes'.
- An unmarked version of your revised paper without tracked changes. You should upload this as a separate file labeled 'Manuscript'.

Guidelines for resubmitting your figure files are available below the reviewer comments at the end of this letter.

As the corresponding author, your ORCID iD is verified in the submission system and will appear in the published article. PLOS supports the use of ORCID, and we encourage all coauthors to register for an ORCID iD and use it as well. Please encourage your coauthors to verify their ORCID iD within the submission system before final acceptance, as unverified ORCID iDs will not appear in the published article. *Only*  the individual author can complete the verification step; PLOS staff *cannot*  verify ORCID iDs on behalf of authors.

We look forward to receiving your revised manuscript.

Kind regards,

Lambert Zixin Li, Ph.D.

Academic Editor

PLOS Mental Health

Journal Requirements:

1. Please update your online Competing Interests statement. If you have no competing interests to declare, please state: "The authors have declared that no competing interests exist."

2. If the reviewer comments include a recommendation to cite specific previously published works, please review and evaluate these publications to determine whether they are relevant and should be cited. There is no requirement to cite these works unless the editor has indicated otherwise.

Additional Editor Comments (if provided):

Dear Authors,

Thank you for submitting your manuscript to PLOS Mental Health. After considering the reviewers’ comments and my own assessment, I would like to invite a major revision. The manuscript addresses an important topic, but substantial concerns remain regarding scientific validity and methodological reproducibility.

Please revise the manuscript carefully to address all reviewer comments and provide a detailed point-by-point response. In particular, the revised version should strengthen the clarity and completeness of the methods, reporting, and analyses so that the study can be evaluated more confidently and reproduced more readily. As this is a cross-sectional study, please ensure that the manuscript follows the STROBE Statement, specifically the checklist for cross-sectional studies.

I look forward to receiving your revised submission.

Best regards,

Lambert Zixin Li, PhD

[Note: HTML markup is below. Please do not edit.]

Reviewers' comments:

Reviewer's Responses to Questions

**Comments to the Author**

1. Does this manuscript meet PLOS Mental Health’s publication criteria?

Reviewer #1: Partly

Reviewer #2: Yes

Reviewer #3: Yes

**********

2. Has the statistical analysis been performed appropriately and rigorously?

Reviewer #1: Yes

Reviewer #2: Yes

Reviewer #3: Yes

**********

3. Have the authors made all data underlying the findings in their manuscript fully available (please refer to the Data Availability Statement at the start of the manuscript PDF file)?

The PLOS Data policy

Reviewer #1: Yes

Reviewer #2: Yes

Reviewer #3: Yes

**********

4. Is the manuscript presented in an intelligible fashion and written in standard English?

Reviewer #1: Yes

Reviewer #2: Yes

Reviewer #3: Yes

**********

Reviewer #1: I recommend this manuscript to be Major Revision, some comments need the author below address:

1. The authors should consistently use causal-neutral language such as "was associated with" rather than "led to," "reduced" or "contributed to".

2. The limitations section should discuss the potential impact of residual confounding more explicitly

3. Please double check, how the complex survey design was incorporated into the regression analyses

4. The lower odds of intending to leave among nurses providing online or blended care may reflect differences in workplace characteristics rather than the delivery modality itself. For example, nurses providing virtual care may have lower patient acuity, different job responsibilities, greater schedule flexibility or better organizational support. These alternative explanations should be acknowledged in the Discussion as well.

5. Please consider including more recent literature (2023-2025) on nurse burnout, workforce retention and post-pandemic implementation of virtual care to better contextualize the findings.

Reviewer #2: Strengths:

1. Large nationally representative dataset.

2. Timely and policy-relevant research question.

3. Appropriate use of multivariable regression.

4. Important workforce mental health outcome.

5. Findings have practical relevance for health workforce planning.

Areas for improvement:

1. The Introduction section is generally well organized. However, more literature on pre-pandemic nurse retention would improve context, and recent post-pandemic workforce evidence could also be incorporated.

2. The authors should better justify the novelty of the study, given their previous publications using the same dataset on physicians and nurses (reference #15 and #16). The manuscript should clearly articulate the additional contribution of focusing specifically on mental health-driven intention to quit.

3. The Methods section:

a. Would benefit from additional detail on the handling of missing data, proportion of missing values, complete-case versus imputation, model diagnostics, assessment of multicollinearity, goodness-of-fit, and whether interaction terms were examined. These are standard expectations for logistic regression reporting.

b. Should explain the very narrow confidence intervals reported for several adjusted odds ratios e.g., Online: OR = 0.49, 95% CI 0.45–0.52; IPC: OR = 2.66, 95% CI 2.57–2.74; and Ontario: OR = 2.75, 95% CI 2.68–2.84. Such precision is unusual given an effective sample of approx. 3,100 nurses.

4. Results are clearly presented.

a. However, the narrative largely repeats Table 2. Consider focusing on strongest predictors, unexpected findings and clinically important associations.

b. Healthcare delivery modality is categorized simply as in-person, online and blended. However, these categories likely encompass tremendous heterogeneity e.g., telephone only, video consultation, asynchronous messaging, occasional virtual clinics and predominantly virtual practice. The manuscript should acknowledge that the exposure is relatively crude.

5. The interpretation of the findings should be more cautious.

a. The study is cross-sectional, causal language should be avoided (e.g., “telemedicine may contribute positively to nurses' experiences” and “blended care contributes to workforce retention”, etc).

b. The possibility of reverse causation (e.g., nurses already experiencing burnout may avoid virtual work) and residual confounding should be discussed more explicitly.

c. Policy recommendations regarding telemedicine and workforce retention should be framed as hypotheses supported by associations rather than causal conclusions.

6. The limitations section should be expanded to acknowledge important unmeasured confounders (e.g., nursing specialty, workload, staffing levels, resilience, and burnout severity, etc) and the inability to establish temporal relationships.

Reviewer #3: The study talks about associations between the mode of employment (remote, hybrid, and presential) and other characteristics, and how nurses wanted to leave the job due to mental health problems. It is an important topic, and the paper was well written. However, I still have some questions to better understand and improve the work.

The title is confusing; I could not understand the paper's message from it. A simpler and more direct title would be better.

There are some acronyms not spelled out in the first appearance.

"A total of 12,246 healthcare workers aged 18 years or older residing in Canada completed the study." So why "This study included 3107 nurses, weighted to represent 353,980 nurses in Canada."

Does Table 1 represent the 353,980 nurses?

**********

what does this mean?). If published, this will include your full peer review and any attached files.

**Do you want your identity to be public for this peer review?**  If you choose “no”, your identity will remain anonymous but your review may still be made public.

For information about this choice, including consent withdrawal, please see our Privacy Policy

Reviewer #1: **Yes:** SAMPHOAS CHIEN

Reviewer #2: No

Reviewer #3: No

**********

[NOTE: If reviewer comments were submitted as an attachment file, they will be attached to this email and accessible via the submission site. Please log into your account, locate the manuscript record, and check for the action link "View Attachments". If this link does not appear, there are no attachment files.]

# Author comment: Author response to Decision Letter 1

**DOI:** 10.1371/journal.pmen.0000692.r002

29 Jul 2026

Attachment Submitted filename: Response to Reviewers.docx

# Peer review: Decision Letter 1

**DOI:** 10.1371/journal.pmen.0000692.r003

**Authors:** Lambert Zixin Li

18 Aug 2026

PMEN-D-26-00345R1

Healthcare Delivery Modality and Mental Health–Related Intention to Leave Their Job Among Nurses in Canada

PLOS Mental Health

Dear Dr. Ali-Hassan,

Thank you for submitting your manuscript to PLOS Mental Health. After careful consideration, we feel that it has merit but does not fully meet PLOS Mental Health’s publication criteria as it currently stands. Therefore, we invite you to submit a revised version of the manuscript that addresses the points raised during the review process.

Please submit your revised manuscript by Sep 17 2026 11:59PM. If you will need more time than this to complete your revisions, please reply to this message or contact the journal office at mentalhealth@plos.org. When you're ready to submit your revision, log on to https://www.editorialmanager.com/pmen/ and select the 'Submissions Needing Revision' folder to locate your manuscript file.

Please include the following items when submitting your revised manuscript:

- A letter that responds to each point raised by the editor and reviewer(s). You should upload this letter as a separate file labeled 'Response to Reviewers'.
- A marked-up copy of your manuscript that highlights changes made to the original version. You should upload this as a separate file labeled 'Revised Manuscript with Track Changes'.
- An unmarked version of your revised paper without tracked changes. You should upload this as a separate file labeled 'Manuscript'.

Guidelines for resubmitting your figure files are available below the reviewer comments at the end of this letter.

As the corresponding author, your ORCID iD is verified in the submission system and will appear in the published article. PLOS supports the use of ORCID, and we encourage all coauthors to register for an ORCID iD and use it as well. Please encourage your coauthors to verify their ORCID iD within the submission system before final acceptance, as unverified ORCID iDs will not appear in the published article. *Only*  the individual author can complete the verification step; PLOS staff *cannot*  verify ORCID iDs on behalf of authors.

We look forward to receiving your revised manuscript.

Kind regards,

Lambert Zixin Li, Ph.D.

Academic Editor

PLOS Mental Health

**Journal Requirements:**

If the reviewer comments include a recommendation to cite specific previously published works, please review and evaluate these publications to determine whether they are relevant and should be cited. There is no requirement to cite these works unless the editor has indicated otherwise.

Please review your reference list to ensure that it is complete and correct. If you have cited papers that have been retracted, please include the rationale for doing so in the manuscript text, or remove these references and replace them with relevant current references. Any changes to the reference list should be mentioned in the rebuttal letter that accompanies your revised manuscript. If you need to cite a retracted article, indicate the article’s retracted status in the References list and also include a citation and full reference for the retraction notice.

**Additional Editor Comments (if provided):**

[Note: HTML markup is below. Please do not edit.]

Reviewers' comments:

Reviewer's Responses to Questions

**Comments to the Author**

Reviewer #1: All comments have been addressed

Reviewer #2: All comments have been addressed

Reviewer #3: All comments have been addressed

**********

publication criteria?

Reviewer #1: Yes

Reviewer #2: Yes

Reviewer #3: Yes

**********

3. Has the statistical analysis been performed appropriately and rigorously?

Reviewer #1: Yes

Reviewer #2: Yes

Reviewer #3: Yes

**********

4. Have the authors made all data underlying the findings in their manuscript fully available (please refer to the Data Availability Statement at the start of the manuscript PDF file)?

The PLOS Data policy

Reviewer #1: Yes

Reviewer #2: Yes

Reviewer #3: Yes

**********

5. Is the manuscript presented in an intelligible fashion and written in standard English?

Reviewer #1: Yes

Reviewer #2: Yes

Reviewer #3: Yes

**********

**Reviewer #1:**  So great that you have improved your manuscript so far, I recommend this manuscript subject to these relatetively minor correction, I believe the manuscript would be suitable for publication. From the minor revision, there a few issues remain before it suitable for accepatance.

1. First, please recheck Table 2, the adjusted estimate for outpatient/ambulatory care (OR = 0.37, 95% CI 0.35–0.38) is reported with p = 0.366; the confidence interval and p-value appear statistically inconsistent.

2. Second, the Discussion states that nurses with “two years of experience or less” had 2.3-fold higher odds of the outcome, whereas the analyzed category is “less than 10 years.” Please correct this discrepancy.

3. Should you add the statements suggesting that virtual care may confer psychological benefits should be further tempered given the cross-sectional design.

**Reviewer #2:**  The authors have responded constructively to the comments and have made several substantive improvements to the manuscript.

However, a few issues remain:

1. The explanation for the unusually narrow confidence intervals is not fully convincing and should be further clarified, particularly regarding the complex survey variance estimation i.e., authors have explained how the CIs were generated but not convincingly why they are so narrow.

2. Although the authors state that causal language has been removed, some causal or causal-suggestive wording remains in the Discussion (e.g., flexible modalities “may confer psychological as well as professional benefits” and that telemedical flexibility “may help buffer” negative impacts and “may potentially contribute” to retention) and Conclusion (telemedicine may “help optimize patient care while contributing to more positive workplace experiences”) and should be revised to reflect the cross-sectional design.

I suggest the authors to explicitly state that the findings generate hypotheses about potentially beneficial workplace characteristics associated with virtual/blended care, rather than demonstrating that changing delivery modality will improve retention.

3. There appear to be serious inconsistencies in Table 2 p-values. For example, the table reports:

a. Outpatient/ambulatory care: adjusted OR 0.37, 95% CI 0.35–0.38, p = 0.366;

b. Other care: adjusted OR 0.99, 95% CI 0.97–1.01, p = 0.990; and

c. Female: adjusted OR 1.04, 95% CI 1.00–1.08, p < 0.001.

These combinations are questionable. In particular, an OR of 0.37 with a 95% CI of 0.35–0.38 cannot plausibly have p = 0.366, because the CI is far from 1. Likewise, OR 0.99 with CI 0.97–1.01 would not normally correspond to p = 0.990. The female estimate also warrants checking. The table should therefore be rechecked against the original statistical output, not merely copy-edited.

**Reviewer #3:**  I thank the authors for their response to the other reviewers and me.

**********

what does this mean?). If published, this will include your full peer review and any attached files.

**Do you want your identity to be public for this peer review?**  If you choose “no”, your identity will remain anonymous but your review may still be made public.

For information about this choice, including consent withdrawal, please see our Privacy Policy

Reviewer #1: **Yes:** SAMPHOAS CHIEN, PhD

Reviewer #2: No

Reviewer #3: No

**********

[NOTE: If reviewer comments were submitted as an attachment file, they will be attached to this email and accessible via the submission site. Please log into your account, locate the manuscript record, and check for the action link "View Attachments". If this link does not appear, there are no attachment files.]

**Figure Resubmissions:**

# Author comment: Author response to Decision Letter 2

**DOI:** 10.1371/journal.pmen.0000692.r004

24 Aug 2026

Attachment Submitted filename: Response_to_Reviewers_auresp_2.docx

# Editor report: Decision Letter 2

**DOI:** 10.1371/journal.pmen.0000692.r005

**Authors:** Lambert Zixin Li

28 Aug 2026

Healthcare Delivery Modality and Mental Health–Related Intention to Leave Their Job Among Nurses in Canada

PMEN-D-26-00345R2

Dear Dr. Ali-Hassan,

We are pleased to inform you that your manuscript 'Healthcare Delivery Modality and Mental Health–Related Intention to Leave Their Job Among Nurses in Canada' has been provisionally accepted for publication in PLOS Mental Health.

Before your manuscript can be formally accepted you will need to complete some formatting changes, which you will receive in a follow up email. A member of our team will be in touch with a set of requests.

Please note that your manuscript will not be scheduled for publication until you have made the required changes, so a swift response is appreciated.

IMPORTANT: The editorial review process is now complete. PLOS will only permit corrections to spelling, formatting or significant scientific errors from this point onwards. Requests for major changes, or any which affect the scientific understanding of your work, will cause delays to the publication date of your manuscript.

If your institution or institutions have a press office, please notify them about your upcoming paper to help maximize its impact. If they'll be preparing press materials, please inform our press team as soon as possible -- no later than 48 hours after receiving the formal acceptance. Your manuscript will remain under strict press embargo until 2 pm Eastern Time on the date of publication. For more information, please contact mentalhealth@plos.org.

Thank you again for supporting Open Access publishing; we are looking forward to publishing your work in PLOS Mental Health.

Best regards,

Lambert Zixin Li, Ph.D.

Academic Editor

PLOS Mental Health

***********************************************************

Reviewer Comments (if any, and for reference):
